# Vaginal Chancres

**Published:** 1882-02

**Authors:** 


### Selections.

### Vaginal Chancres.

Dr. M. P. Binet reports two cases of true vaginal chancre. The first was a girl of 18, who showed an erosive syphiloderm of the fossa navicularis, slight swelling of the right labium majus, which was more red than the other, and multiple adenitis of the right inguinal region. On examination, the meatus of the cervix uteri was slightly open and eroded; there was double adeno-lymphitis toward the lateral culs-de-sac on the sides of the uterus. On the right vaginal wall, at the junction of the upper and middle third, the finger could perceive a depressed erosion, rounded in contour, sharply circumscribed, not painful. On examination with the speculum, the cervix showed follicular erosion, together with the glutinous mucous discharge of metritis. The vaginal erosion showed all the characteristic signs of indurated chancre, the floor red, smooth, shining, non-purulent; the edges slightly elevated, and passing without a ridge into the floor of the erosion and into the surrounding tissues, of which the color was normal; not excavated or everted. The lesion was about the size of a ten-cent piece, and was seated upon the right vaginal wall, near the inferior extremity of the os uteri. It was difficult to make out the induration, on account of the laxity of the vaginal walls and the distance of the lesion from the vulvar ring. However, in passing the finger lightly over the surface in the neighborhood of the erosion, a slight resistance could be perceived, as of a more resilient surface. In addition, by passing two fingers deeply into the vagina a foliaceous induration could be perceived. The lymphatics running along the walls of the vagina were enlarged; they appeared to leave the erosion and run toward the indurated post-pubic ganglia. Small ganglia could likewise be perceived in the neighborhood of the obturator foramen. On the body an eruption of roseola, with some circular erythematous patches and discrete squamous papules, could be perceived. Under a mercurial and tonic treatment the various symptoms, including the chancre itself, disappeared without any local applications having been made.

Binet's second case was that of a girl of 19, who entered the hospital for erosions about the labia and anus. To the touch there could be perceived on the posterior wall of the vagina, just within the carunculæ myrtiformes, an eroded, non-painful, finely rugous surface, of which the floor gave the sensation cf greater resiliency. On ocular examination, the lesion presented the characteristics of chancre. Its floor was grayish, the edges slightly raised, not everted or excavated, and passing imperceptibly and without a ridge into the surrounding tissues. The lesion was roundish in shape, and was the size of a quarter-dollar. Parchment induration under and around the sore could be distinctly made out with the fingers, permitting it to be enucleated, so to speak, from the surrounding tissues. There was some vaginities in the culs-de-sac, and there were also follicular erosions about the os uteri, with the muco-glutinous discharge of metritis. No eruption on the general surface. The patient was under observation for two weeks, during which time (probably under the influence of treatment, though this is not stated) the chancre became almost completely cicatrized, presenting a smooth, pale, violaceous tint, with a slightly depressed surface.

In discussing the causes of the extreme rarity of chancre of the vagina, Binet attributes this to the fact that the virus must either be deposited in some abrasion or in a follicle. But the vaginal mucous membrane, with its thick layers of epithelium, is extremely resistant, and is rarely eroded in sexual intercourse. Moreover, it has no open glandular orifices. Vaginal chancre is extremely rare. Fournier, in 249 chancres of the female genital organs, saw only one in the vagina, and that doubtful. Binet, in 128 chancres of the female genitalia in Martineau's wards at the Lourcine, only observed the two above given. No other observers have reported the lesion.—*La France Med.*

